# Oral Administration of MVA-Vectored Vaccines Induces Robust, Long-Lasting Neutralizing Antibody Responses and Provides Complete Protection Against SARS-CoV-2 in Mice, Minks, and Cats

**DOI:** 10.3390/vaccines13121207

**Published:** 2025-11-29

**Authors:** Linya Feng, Hong Huo, Yunlei Wang, Lei Shuai, Gongxun Zhong, Zhiyuan Wen, Liyan Peng, Jinying Ge, Jinliang Wang, Chong Wang, Weiye Chen, Xijun He, Xijun Wang, Zhigao Bu

**Affiliations:** 1 State Key Laboratory for Animal Disease Control and Prevention, Harbin Veterinary Research Institute, Chinese Academy of Agricultural Sciences, Harbin 150069, China; fenglinya@mail.kiz.ac.cn (L.F.); huohong617@163.com (H.H.); wangyunlei2023@163.com (Y.W.); shuailei@caas.cn (L.S.); zhonggongxun@caas.cn (G.Z.); wenzhiyuan@caas.cn (Z.W.); pengliyan0811@163.com (L.P.); gejinying@caas.cn (J.G.); wangjinliang@caas.cn (J.W.); wangchong@caas.cn (C.W.); chenweiye@caas.cn (W.C.); 2 National High Containment Facilities for Animal Diseases Control and Prevention, Harbin Veterinary Research Institute, Chinese Academy of Agricultural Sciences, Harbin 150069, China; hexijun@caas.cn; 3 Jiangsu Co-Innovation Center for Prevention and Control of Important Animal Infectious Diseases and Zoonoses, Yangzhou University, Yangzhou 225009, China

**Keywords:** COVID-19, SARS-CoV-2, S protein, MVA, vaccine

## Abstract

Background/Objectives: Severe acute respiratory syndrome coronavirus 2 (SARS-CoV-2) can naturally infect a broad spectrum of animal species, with cats, minks, and ferrets being highly susceptible. There is a potential risk that infected animals could transmit viruses to humans. Moreover, SARS-CoV-2 continues to evolve via mutation and genetic recombination, resulting in the continuous emergence of new variants that have triggered a wave of reinfection. Therefore, safe and effective corona virus disease 2019 (COVID-19) vaccines for animals are still being sought. Methods: We generated three recombinant Modified vaccinia virus Ankara (MVAs) expressing the prefusion-stabilized S proteins, S6P, DS6P, and BA2S6P, targeting the full-length S protein genes of the ancestral, Delta, and Omicron BA.2 strains of SARS-CoV-2. Subsequently, the safety, immunogenicity, and protective efficacy of these MVA-based oral COVID-19 vaccine candidates were assessed in mice, minks, and cats. Results: These recombinant MVAs are safe in mice, minks, and cats. Oral or intramuscular vaccination with rMVA-S6P induced a robust SARS-CoV-2 neutralizing antibody (NA) response and conferred complete protection against the SARS-CoV-2 challenge in mice. Meanwhile, oral or intramuscular administration of these recombinant MVAs in combination induced a potent and durable NA response against homotypic SARS-CoV-2 pseudovirus in mice, minks, and cats, respectively. Conclusions: These findings suggest that the MVA-vectored vaccines are promising oral COVID-19 vaccine candidates for animals, and that the combined vaccination approach is an effective administration strategy for such vaccines.

## 1. Introduction

Corona virus disease 2019 (COVID-19), caused by severe acute respiratory syndrome coronavirus 2 (SARS-CoV-2), has been a serious global public health security concern over the past few years [1]. Although global reports of new COVID-19 cases and deaths have decreased since 2023, they remain considerable. SARS-CoV-2 can naturally infect a broad spectrum of animal species, with cats, minks, and ferrets being highly susceptible [1,2,3,4]. Infected animals may serve as intermediate hosts for the cross-species transmission of the virus between humans and animals, as evidenced by human cases of COVID-19 resulting from exposure to infected minks [5,6]. Moreover, the persistent evolution of SARS-CoV-2 via mutation and genetic recombination gives rise to new variants, which have triggered a wave of reinfection [7,8]. Therefore, safe and effective COVID-19 vaccines for humans and animals are still being sought.

A range of COVID-19 vaccines have been licensed for use in the humans, encompassing inactivated, viral-vectored, mRNA, DNA, and subunit vaccines [9,10,11,12,13,14]. All these strategies require intramuscular, subcutaneous, or intranasal vaccination to induce favorable immune responses. However, oral immunization is an effective approach for significantly improving vaccination coverage among hard-to-reach populations such as free-roaming pets, aggressive farmed animals, and wild animals. Experience accumulated over recent decades in rabies control throughout North America and Europe provides compelling evidence that oral vaccination is the most effective strategy for managing infectious diseases in stray, free-ranging, and wild animals. [15]. Therefore, a safe and effective oral COVID-19 vaccine for susceptible animals remains urgently needed.

Modified vaccinia virus Ankara (MVA) is a replication-deficient vaccinia virus and a licensed vaccine for smallpox and monkeypox, incapable of producing progeny viruses in humans and most other mammalian cells [16,17]. It exhibits outstanding safety and can efficiently express exogenous genes, eliciting potent humoral and cellular immune responses in humans and other mammals [18,19]. Therefore, it was widely employed as a vaccine vector for the developing recombinant vaccines against infectious diseases and cancers [20,21,22,23,24,25,26,27,28,29,30,31,32]. Here, we designed and chemically synthesized three prefusion-stabilized modified S protein genes, targeting the full-length S genes of the ancestral, Delta, and Omicron BA.2 strains of SARS-CoV-2. Subsequently, we generated three recombinant MVAs expressing these modified proteins individually. Then, we assessed their potential as live-virus-vectored oral COVID-19 vaccines for animal use.

## 2. Materials and Methods

### 2.1. Viruses and Cells

BHK-21 (ATCC, CCL-10) and Vero E6 (ATCC, CRL-1586) cells were maintained in Dulbecco’s modified eagle’s medium (DMEM) supplemented with 5% and 10% fetal calf serum (FCS), respectively. Chicken fibroblast (DF-1) cells (ATCC, CRL-3586) were cultured in DMEM/F12 medium containing 10% FCS. Primary chicken embryo fibroblasts (CEFs), prepared from 10-day-old fertile eggs, were grown in DMEM with 10% FCS. MVA and rMVA-eGFP/mCherry, which co-expressing green and red fluorescent proteins (GFP and mCherry), came from our laboratory and were propagated in primary CEFs. The recombinant virus rMVA-eGFP/mCherry was generated by integrating expression cassettes for EGFP and mCherry into the *Eco*R I site of the TK gene locus in the MVA genome via homologous recombination. The SARS-CoV-2 strain HRB25 and HRB26M were obtained from the National High Containment Facilities for Animal Diseases Control and Prevention, Harbin Veterinary Research Institute (HVRI), Chinese Academy of Agricultural Sciences (CAAS) (Harbin, China). HRB25 was originally isolated from a patient in Harbin in February 2020. The HRB26M strain is a mouse-adapted variant derived from the clinical isolate HRB26 through serial passage in BALB/c mice. HRB26M belongs to an ancestral strain lineage and efficiently replicates in both the upper and lower respiratory tracts of both BALB/c and C57BL/6J mice. Both SARS-CoV-2 strains were propagated and titrated in Vero E6 cells. Three vesicular stomatitis virus (VSV)-based SARS-CoV-2 pseudoviruses (VSVΔG-SARS-CoV-2S-eGFP, VSVΔG-DeltaS-eGFP, and VSVΔG-OmicronS-eGFP) came from our laboratory. In these recombinant viruses, the native VSV glycoprotein (G) gene is replaced with the S gene of the SARS-CoV-2 ancestral, Delta, or Omicron BA.2 strains, respectively, and each pseudovirus concomitantly expressing GFP. These SARS-CoV-2 pseudoviruses were propagated and titrated in Vero E6 cells.

### 2.2. Redesign and Immunogenicity of the Prefusion-Stabilized S Proteins

We designed and chemically synthesized three prefusion-stabilized S genes S6P, DS6P, and BA2S6P, targeting the full-length S genes of the ancestral (GenBank accession No. NC045512.2), Delta (GenBank accession No. MZ702566.1), and Omicron BA.2 (GenBank accession No. OM837877.1) strains of SARS-CoV-2 in Comate Bioscience Co., Ltd. (Changchun, China). The modifications of these modified S genes involve codon optimization for mammalian usage, replacement of the tissue plasminogen activator signal peptide gene (*tPASP*), furin recognition sites substitutions (aa 682–685 RRAR-GSAS), and six proline (6P) substitutions (F817P, A892P, A899P, A942P, K986P, V987P) as described previously [33]. Subsequently, the DNA of S6P, DS6P, or BA2S6P was inserted into the plasmid vector pCAGGS under the control of the chicken beta-actin promoter to construct the recombinant plasmids, named pCAGG-S6P, pCAGG-DS6P, and pCAGG-BA2S6P. For manufacturing COVID-19 DNA vaccines, these recombinant plasmids were packaged into lipid nanoparticles (LNPs) using a lipid mixture comprising an ionizable lipid, DSPC, cholesterol, ethanol, etc., via a micro-channel device at Zhongqi Pharmaceutical Technology Co., Ltd. of CSPC Pharmaceutical Group (Shijiazhuang, China). Further, to assess the immunogenicity and duration of immunity conferred by these modified proteins either individually or in combination, five groups of six 8-week-old BALB/c mice were inoculated intramuscularly (i.m.) with 0.1 mL 30 µg of pCAGGS or these DNA vaccines individually or with 0.1 mL of the trivalent DNA vaccine containing 30 µg of each NDA vaccine. At 4 weeks post-vaccination (p.v.), the mice received a second vaccine dose, respectively. The mice were monitored daily for disease symptoms and mortality for 54 weeks. Serum samples were collected from all mice before vaccination and at various time points p.v. for SARS-CoV-2 neutralizing antibody (NA) assessment.

### 2.3. Construction of Recombinant Viruses

The DNA of S6P, DS6P, or BA2S6P was individually cloned into the *Eco*R I site of the transfer vector pCI-MVATK, thereby placing them under the control of the vaccinia virus early/late promoter H6. These recombinant transfer vectors were designated pCI-MVATK-S6P, pCI-MVATK-DS6P, and pCI-MVATK-BA2S6P, respectively. To generate recombinant MVAs, the respective recombinant transfer vector was transfected into BHK-21 cells pre-infected with rMVA-eGFP/mCherry, allowing recombination into the *Eco*R I site of the TK gene in the MVA genome. The recombinant MVAs were clonally purified by successive rounds of non-fluorescent foci isolation in DF-1 cells and propagated in primary CEFs. The purity of recombinant MVAs was confirmed by PCR amplification. The recombinant MVAs were designated as rMVA-S6P, rMVA-DS6P, and rMVA-BA2S6P, respectively.

### 2.4. Confirmation of the SARS-CoV-2 S Gene Expression in Cells Infected with Recombinant MVAs

The expression of the SARS-CoV-2 S protein by the recombinant MVAs was confirmed using both immunofluorescence assay (IFA) and Western blotting. For IFA, DF-1 cells in 24-well plates were infected with MVA and the rescued recombinant MVAs at a multiplicity of infection (MOI) of 0.02 and incubated for 36 h. Then, cells were probed with a 1:100 dilution of rabbit anti-SARS-CoV-2 Spike/RBD polyclonal antibody (Sino Biological Inc., Beijing, China), followed by an FITC-conjugated goat anti-rabbit secondary antibody (Sigma, Darmstadt, Germany). Imaging was performed using an EVOS FL cell imaging system with IMAGE J 1.54d software (Thermo Fisher Scientific). For Western blotting, DF-1 cells in 6-well plates were infected with MVA and the rescued recombinant MVAs at an MOI of 0.02 and incubated for 36 h. Lysates from infected cells were subjected to SDS-PAGE (10% gel) under denaturing conditions. The membranes were incubated with the same primary antibody as used in IFA (1:100) and an infrared dye-labeled donkey anti-rabbit IgG secondary antibody (LI-COR Biosciences, Lincoln, Nebraska, USA). Protein bands were visualized and analyzed using an Odyssey CLx Imaging System with Image Studio 5.2.5 software (LI-COR Biosciences).

### 2.5. Growth Properties of the Recombinant MVAs

DF-1 cells in 12-well plates were inoculated with MVA and the recombinant MVAs at an MOI of 0.02. Following a 1 h adsorption period at 37 °C, the inoculum was removed, and the cells were washed twice with PBS before being maintained in DMEM/F12 medium supplemented with 2% FCS at 37 °C under 5% CO_2_. Cells and supernatants were harvested at various time points post-inoculation (p.i.) and stored at −80 °C. After the cells and supernatants were frozen and thawed three times, the recombinant MVAs were titrated on DF-1 cells in 96-well plates.

### 2.6. Ethics Statement

All animal studies were performed in strict accordance with the Guidelines for the Care and Use of Laboratory Animals issued by the Ministry of Science and Technology of China and were approved by the Animal Ethics Committee of the HVRI, CAAS. All experiments involving live SARS-CoV-2 were performed in a Biosafety Level 4 (BSL-4) or Animal Biosafety Level 4 (ABSL-4) facility, while all other animal immunization work was conducted under ABSL-2 conditions.

### 2.7. Pathogenicity in Mice

Seventy-two 6-week-old female BALB/c mice were obtained from Vital River (Beijing, China) and randomly assigned to eight groups (*n* = 9 per group). Eight groups of mice were inoculated intramuscularly (i.m.) or orally (administered dropwise into the oral cavity) with 1 × 10^7^ TCID_50_ (in 0.1 mL PBS) of MVA, rMVA-S6P, rMVA-DS6P, and rMVA-BA2S6P, respectively. On days 3, 5, and 7 p.i., we euthanized three mice per group and collected their organs (brain, lung, liver, spleen, kidney, and heart). Each organ was homogenized in 0.5 mL PBS. The viral titers in these homogenates were then determined in DF-1 cells, with each sample tested in triplicate. The lower limit of detection is 3.16 TCID_50_/mL.

### 2.8. Immunization Study

Building on the range of doses (e.g., 1 × 10^7^ PFU to 1 × 10^8^ PFU) previously employed for intramuscular immunization with MVA-based COVID-19 vaccines in mice [34,35,36,37], the present study adopted a dose of 1 × 10^7^ TCID_50_ for recombinant MVAs. Accordingly, based on our prior findings with oral rabies vaccines demonstrating that a 5- to 10-fold higher dose than the intramuscular route is necessary to induce effective immunity, an oral dose of 5 × 10^7^ TCID_50_ was selected.

Seventy-two 8-week-old female BALB/c mice were purchased from Vital River (Beijing, China). These mice were randomly divided into twelve groups (*n* = 6 per group). Two groups were inoculated i.m. with 1 × 10^7^ TCID_50_ of rMVA-S6P in 0.1 mL PBS or orally (administered dropwise into the oral cavity) with 5 × 10^7^ TCID_50_ of rMVA-S6P in 0.1 mL PBS. A third group was inoculated i.m. and orally with 1 × 10^7^ TCID_50_ and 5 × 10^7^ TCID_50_ of MVA, both in 0.1 mL PBS. The mice were observed daily for body weight changes for 3 weeks and signs of disease or death for 6 weeks. Additionally, to assess the safety, immunogenicity, and duration of immunity elicited by individual or combined recombinant MVA vaccines, nine groups of mice were inoculated i.m. or orally with PBS, 1 × 10^7^ TCID_50_ of MVA, 1 × 10^7^ TCID_50_ of individual recombinant MVAs, or a trivalent vaccine containing 1 × 10^7^ TCID_50_ of each recombinant MVA, all delivered in a 0.1 mL PBS volume. We observed the mice daily for clinical signs of disease or death for 54 weeks and recorded body weight for the first 3 weeks.

Given that minks and cats have a much larger body weight than mice, we therefore selected an oral dose 10 times higher than the murine dose for these animals. Ten 6-month-old farmed minks (four males and six females) and ten 6-month-old domestic cats (three males and seven females) were enrolled in this study. All animals, sourced from local farms, were confirmed seronegative for SARS-CoV-2 by a virus neutralization test before immunization. Following random allocation into two groups per species (*n* = 5), one group of minks and one group of cats received an oral inoculation (administered dropwise onto the center of the tongue) of 5 × 10^8^ TCID_50_ MVA in 1 mL PBS. The other group of minks and the other group of cats received the trivalent vaccine (1 mL PBS containing 5 × 10^8^ TCID_50_ of each recombinant MVA), administered orally in the same manner. Minks were monitored daily for clinical signs or mortality for 9 weeks, while cats were observed for 14 weeks.

At 3 weeks post-vaccination (p.v.), all animals were boosted with the same vaccine, respectively. To assess SARS-CoV-2 NAs, serum samples were collected prior to vaccination and at different time points p.v.

### 2.9. Challenge Test

At 7 weeks p.v., three groups of mice vaccinated with either MVA or rMVA-S6P were challenged intranasally (i.n.) with 10^3.6^ PFU of strain HRB26M in 100 µL volume. On days 3 and 5 post-challenge (p.c.), the nasal turbinates and lungs were collected from three mice in each group and homogenized for viral RNA detection by qPCR and virus titration. The viral RNA detection and virus titration were performed as described previously [1,2].

### 2.10. Serological Tests

Two-fold serial dilutions of sera were incubated with 100 PFU of either SARS-CoV-2 strain HRB25 or a homotypic SARS-CoV-2 pseudovirus for 1 h at 37 °C. The neutralization mixture was then subjected to a plaque assay on Vero E6 cells. The NA titer was defined as the maximum serum dilution that resulted in a 50% reduction in plaque count compared to the control serum from uninfected animals.

### 2.11. Statistical Analysis

The data were analyzed using GraphPad Prism 8.0.1 (244) software (GraphPad Software Inc., San Diego, CA, USA). Statistical comparisons between the assay results were performed using a two-way ANOVA test or a one-way ANOVA test. *p*-Values < 0.05 were considered statistically significant. *, *p* < 0.05; **, *p* < 0.01.

## 3. Results

### 3.1. COVID-19 DNA Vaccines Expressing S6P, DS6P, and BA2S6P Proteins Are Highly Immunogenic in Mice

**Figure 1:**
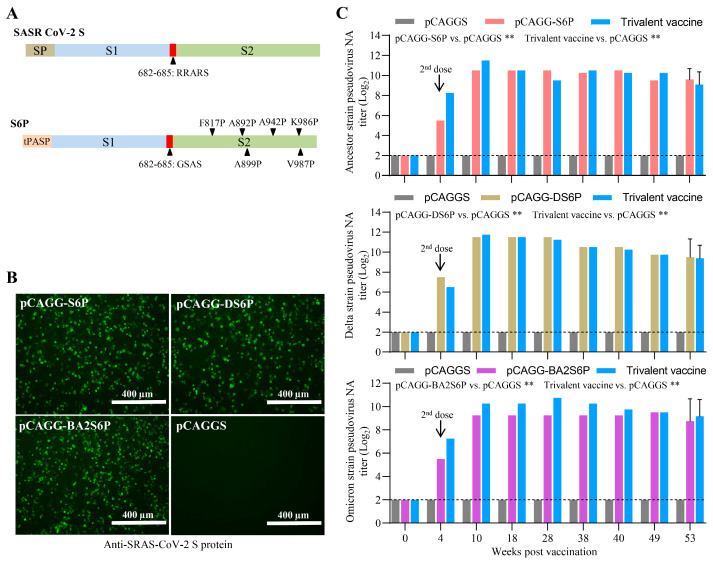
Design and immunogenicity of the modified SARS-CoV-2 S proteins. (**A**) Schematic for designing the prefusion-stabilized S protein gene S6P, targeting the full-length S protein of the ancestral strain of SARS-CoV-2. Modifications of S6P protein gene included codon optimization, furin recognition site substitution, tPASP replacement, and six proline substitutions (6P). (**B**) Detection of S protein expression in recombinant plasmids-transfected BHK-21 cells by using IFA. Scale bar (white line), 400 μm. (**C**) The NA titers against SARS-CoV-2 induced by the DNA vaccines in mice. BALB/c mice (*n* = 6) were initially inoculated and boosted 4 weeks later with 30 µg of three DNA vaccines individually or the trivalent DNA vaccine containing 30 µg of each DNA vaccine. At the indicated time points, sera were collected and pooled for the detection of NA against pseudovirus of the SARS-CoV-2 ancestral strain, Delta strain, or Omicron BA.2 strain. Data are presented as mean ± SD (*n* = 6) at 53 weeks p.v. and individual values (*n* = 1, sera pool) at other time points and were normally distributed (Shapiro–Wilk, *p* > 0.05). A one-way ANOVA followed by Tukey’s multiple comparison test was used. ** *p* < 0.01.

Three modified SARS-CoV-2 S genes, S6P, DS6P, and BA2S6P, were chemically synthesized by Comate Bioscience Co., Ltd. As illustrated in Figure 1A, modifications to these variants include codon optimization for mammalian usage, replacement of the tPASP, removal of the furin recognition sites, and the introduction of six proline (6P) substitutions. Next, the DNA of S6P, DS6P, and BA2S6P was inserted into the plasmid vector pCAGGS to construct the recombinant plasmids named pCAGG-S6P, pCAGG-DS6P, and pCAGG-BA2S6P, respectively. Subsequently, the S protein expression by these recombinant plasmids was confirmed by IFA (Figure 1B) with a rabbit anti-SARS-CoV-2 Spike/RBD polyclonal antibody. Furthermore, the immunogenicity and duration of immunity of these DNA vaccines, alone or in combination, were assessed in mice using the immunization schedules detailed in Section 2. All immunized mice remained healthy. We then tested the NA titers against the homotypic SARS-CoV-2 pseudovirus from the pooled sera of the mice. As shown in Figure 1C, these DNA vaccines, alone or in combination, induce a robust and long-lasting neutralizing antibody response against homotypic SARS-CoV-2 pseudovirus in mice. No significant differences in SARS-CoV-2 NA titers were observed between the groups inoculated with the individual recombinant rMVAs and the trivalent vaccine group. These results indicated that the modified proteins S6P, DS6P, and BA2S6P are immunogenic in mice.

### 3.2. Generation of Recombinant MVAs Expressing the S Genes of SARS-CoV-2

**Figure 2:**
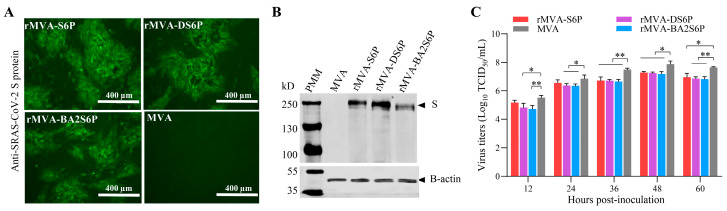
Generation of the recombinant MVAs expressing modified SARS-CoV-2 S genes. (**A**) IFA detection of S protein expression in DF-1 cells infected with recombinant MVAs. Scale bar (white line), 400 μm. (**B**) Western blot analysis of SARS-CoV-2 S protein expressed by recombinant MVAs in DF-1 cells. PMM: Protein molecular weight marker. (**C**) In vitro growth kinetics of recombinant MVAs. DF-1 cell monolayers were infected with MVA and individual recombinant MVAs (MOI = 0.02) and viruses were harvested at the indicated time points. The virus titers (log_10_ TCID_50_/mL; mean ± SD, *n* = 3) are shown. Data were normally distributed (Shapiro–Wilk, *p* > 0.05). Significant differences between groups were determined by employing a two-way ANOVA followed by Tukey’s multiple comparison test. * *p* < 0.05, ** *p* < 0.01.

To generate the recombinant MVAs expressing the modified S proteins, BHK-21 cells pre-infected with rMVA-eGFP/mCherry were individually transfected with the recombinant transfer vectors pCI-MVATK-S6P, pCI-MVATK-DS6P, and pCI-MVATK-BAS6P using the liposome transfection method. The pure recombinant MVAs were obtained by three consecutive rounds of non-fluorescent foci isolation in DF-1 cells. The purity of recombinant MVAs was confirmed by PCR amplification. The recombinant viruses were designated rMVA-S6P, rMVA-DS6P, and rMVA-BA2S6P, respectively. We next investigated if the inserted S gene could be expressed in the recombinant MVA-infected cells. As expected, MVA-infected cells were not stained by rabbit anti-SARS-CoV-2 Spike/RBD polyclonal antibodies (Figure 2A). In contrast, cells infected with rMVA-S6P, rMVA-DS6P, or rMVA-BA2S6P were stained by rabbit polyclonal antibody against SARS-CoV-2 Spike/RBD (Figure 2A). Western blot analysis further verified the successful expression of the S protein expression by rMVA-S6P, rMVA-DS6P, and rMVA-BA2S6P (Figure 2B).

We next compared the growth kinetics of the recombinant viruses with wild-type MVA in DF-1 cells. The rMVA-S6P, rMVA-DS6P, rMVA-BA2S6P, and MVA reached peak titers of 7.29 log_10_, 7.24 log_10_, 7.18 log_10_, and 7.87 log_10_ TCID_50_/mL at 48 h p.i. (Figure 2C). The titers of these recombinant MVAs were slightly lower than that of the parental MVA. Furthermore, the genetic stability of the recombinant MVAs, rMVA-S6P, rMVA-DS6P, and rMVA-BA2S6P, were evaluated through serially passaged 10 times in CEF cells, respectively. The presence and expression of the S gene in the 10th passage of the recombinant MVAs were confirmed by PCR and immunofluorescence.

### 3.3. Introduction and Expression of SARS-CoV-2 S Genes Do Not Increase the Virulence of the MVA Vector in Mice

**Figure 3:**
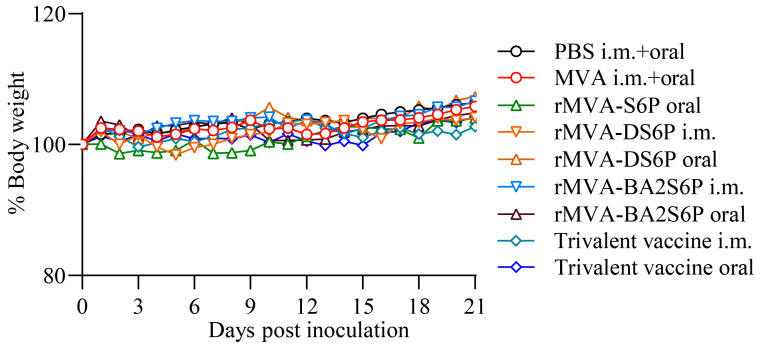
Weight changes in mice inoculated with the recombinant MVAs. BALB/c mice (*n* = 6) were inoculated orally or i.m. with PBS, 1 × 10^7^ TCID_50_ of MVA, rMVA-S6P, rMVA-DS6P, rMVA-BA2S6P, or the trivalent vaccine containing 1 × 10^7^ TCID_50_ of each recombinant MVA. The mice were observed and weighed daily for 21 days. Body weight changes for each group are shown as ratios of the body weight at day 0, which was set as 100. Data were normally distributed (Shapiro–Wilk, *p* > 0.05). Significant differences between groups were determined by employing a one-way ANOVA followed by Tukey’s multiple comparison test.

To assess the replication capacity and pathogenicity of the recombinant MVAs in vivo, mice were inoculated i.m. or orally with PBS, MVA, rMVA-S6P, rMVA-DS6P, rMVA-BA2S6P, and the trivalent vaccine, according to the inoculation schedules detailed in Section 2.7, respectively. Throughout the study, all mice remained healthy, and body weight loss did not exceed 2%. Moreover, no significant differences in body weight changes were observed between the recombinant rMVAs groups and the MVA control group (Figure 3). No viruses were recovered from the heart, liver, spleen, lung, kidney, or brain, which were collected at 3, 5, and 7 days post-infection (p.i.) with 1 × 10^7^ TCID_50_ MVA or recombinant MVAs via i.m. or oral route. These results indicate that the insertion and expression of these SARS-CoV-2 S genes did not enhance the virulence of the vector virus MVA.

### 3.4. Recombinant Virus rMVA-S6P Elicits a Robust NA Response and Provides Complete Protection Against the SARS-CoV-2 Challenge in Mice

**Figure 4:**
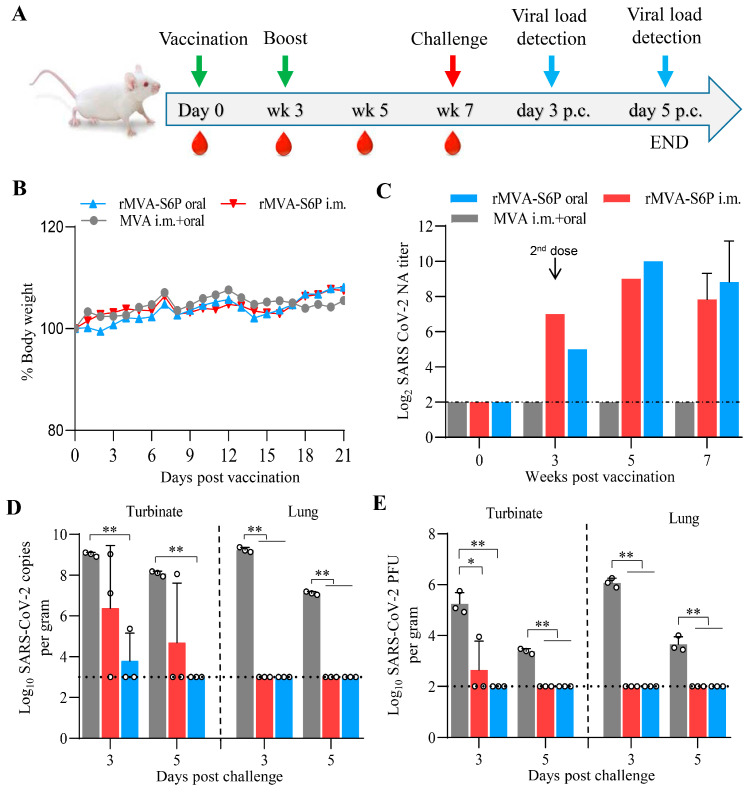
Immunogenicity and protective efficacy of rMVA-S6P in mice. BALB/c mice (*n* = 6) were initially inoculated and boosted orally with 5 × 10^7^ TCID_50_ or i.m. with 1 × 10^7^ TCID_50_ of rMVA-S6P or MVA at 0 time and 3 weeks p.v., with blood collection on days 0 and week 3, 5, and 7 until challenge in week 7 (**A**). Weight changes in mice inoculated with the rMVA-S6P (**B**). The mice were observed and weighed daily for 21 days. Body weight changes for each group are shown as ratios of the body weight at day 0, which was set as 100. At the indicated time points, sera were collected and pooled for the detection of NA against the live SARS-CoV-2. Data are presented as individual values (*n* = 1, sera pool) at weeks 0, 3, and 5 p.v. and mean ± SD (*n* = 6) at 7 weeks p.v. (**C**). At 7 weeks p.v., mice inoculated with rMVA-S6P or MVA were challenged i.n. with 10^3.6^ PFU of SARS-CoV-2 strain HRB26M. On day 3 and day 5 p.c., the nasal turbinates and lungs were collected and homogenized for viral RNA detection by qPCR and virus titration in Vero E6 cells. Viral RNA copies (**D**) and viral titers (**E**) of the challenge virus SARS-CoV-2 in their nasal turbinates and lungs were shown. Data were normally distributed (Shapiro–Wilk, *p* > 0.05). Significant differences between groups were determined by employing a two-way ANOVA followed by Tukey’s multiple comparison test for viral load, and a one-way ANOVA followed by Tukey’s multiple comparison test for NA titers. * *p* < 0.05, ** *p* < 0.01.

Groups of mice (*n* = 6) were inoculated i.m. with 1 × 10^7^ TCID_50_ or orally with 5 × 10^7^ TCID_50_ of MVA or rMVA-S6P (Figure 4A). Throughout the study, no clinical signs of disease were observed in any of the mice. There were no significant differences in body weight changes among the three groups. (Figure 4B). We then tested the NA titers against live SARS-CoV-2 from the pooled sera of the mice. As shown in Figure 4C, SARS-CoV-2 NA was detected in mice inoculated with rMVA-S6P, but not MVA. The titers of SARS-CoV-2 NA were 7 log_2_ and 5 log_2_ in intramuscular and oral groups inoculated with rMVA-S6P at 3 weeks p.v., respectively. At 2 weeks after boosting, the titers of SARS-CoV-2 NA reached a peak of 9 log_2_ (i.m.) and 10 log_2_ (oral), and then declined to 7.83 log_2_ and 8.83 log_2_ at 7 weeks p.v. No significant difference in SARS-CoV-2 NA titers was observed between the two rMVA-S6P immunization routes.

At 7 weeks p.v., groups of six mice inoculated i.m. or orally with rMVA-S6P or MVA were challenged with 10^3.6^ PFU of SARS-CoV-2 strain HRB26M. As shown in Figure 4D,E, high levels of viral RNA and infectious viruses were detected in the turbinates and lungs of MVA-vaccinated mice on days 3 and 5 p.c. In contrast, no viral RNA or infectious virus was detected in the turbinates or lungs of any orally vaccinated rMVA-S6P mice, except for a low level of viral RNA detected in the turbinates of 1/3 mice on day 3 p.c. Meanwhile, in the rMVA-S6P-muscularly vaccinated mice, no viral RNA and infectious virus was detected in the lungs of any mice on days 3 and 5 P.c. In the turbinate, viral RNA was detected in two of three mice, and infectious virus at low titer in one mouse on day 3 p.c. By day 5 p.c., viral RNA was detected in only one mouse, and no infectious virus was detected in all three mice.

### 3.5. Combined Immunization with These Recombinant MVAs Did Not Compromise the Immunogenicity of Each Recombinant MVA in Mice

**Figure 5:**
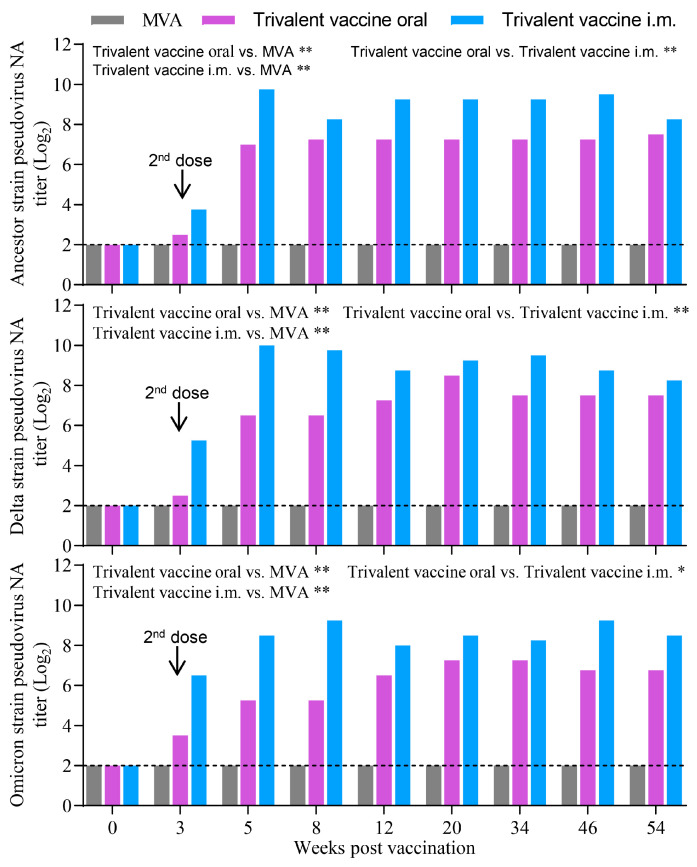
Immunogenicity and duration of immunity of the trivalent vaccine in mice. BALB/c mice (*n* = 6) were initially inoculated i.m. or orally and boosted 3 weeks later with the trivalent vaccine containing 1 × 10^7^ TCID_50_ of each recombinant MVA or 1 × 10^7^ TCID_50_ of MVA, respectively. At the indicated time points, sera were collected and pooled for the detection of NA against pseudovirus of the SARS-CoV-2 ancestral strain, Delta strain, or Omicron BA.2 strains. Data are presented as individual values (*n* = 1, sera pool). Data were normally distributed (Shapiro–Wilk, *p* > 0.05). Significant differences between groups were determined by employing a one-way ANOVA followed by Tukey’s multiple comparison test. * *p* < 0.05, ** *p* < 0.01.

To investigate the immunogenicity of combined immunization with recombinant viruses, three groups of mice were inoculated i.m. or orally with MVA or the trivalent vaccine. We then tested the NA titers against the homotypic pseudovirus from the pooled sera of the mice. As shown in Figure 5, SARS-CoV-2 NA was detected in the trivalent vaccine groups, but not the MVA group. The titers of NA against ancestral, Delta, and Omicron strains were 3.75 log_2_, 5.25 log_2_, and 6.50 log_2_ or 2.50 log_2_, 2.50 log_2_, and 3.50 log_2_ in intramuscular or oral groups at 3 weeks p.v., respectively. At two weeks after the second dose, the titers of NA against ancestral, Delta, and Omicron strains for the intramuscular group reached a peak of 9.75 log_2_, 10.00 log_2_, and 9.25 log_2_, respectively. In contrast, the oral group reached peak titers of 7.50 log_2_, 8.50 log_2_, and 7.25 log_2_ at 20 weeks p.v., respectively. Interestingly, the titers of NA against ancestral, Delta, and Omicron strains still reached 8.25 log_2_, 8.25 log_2_, and 8.50 log_2_ or 7.25 log_2_, 7.50 log_2_, and 6.75 log_2_ in the intramuscular or oral groups at 54 weeks p.v., respectively. Overall, the intramuscular group developed significantly higher levels of NA response compared to the oral group. All immunized mice remained healthy, and no abnormal dietary behaviour or other clinical abnormalities were observed throughout the study. These results demonstrate that the trivalent vaccine is immunogenic in mice.

### 3.6. Oral Vaccination with the Trivalent Vaccine Induced a Robust Neutralizing Antibody Response Against SARS-CoV-2 Pseudovirus in Minks and Cats

**Figure 6:**
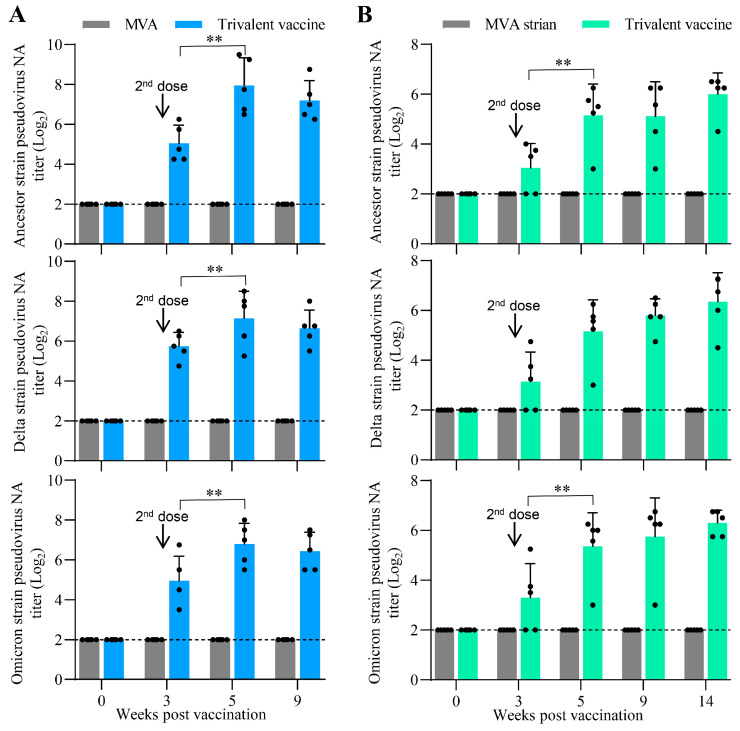
Immunogenicity of the trivalent vaccine in minks and cats. Minks (*n* = 5) (**A**) and cats (*n* = 5) (**B**) were initially inoculated orally and boosted 3 weeks later with 5 × 10^8^ TCID_50_ of MVA or the trivalent vaccine containing 5 × 10^8^ TCID_50_ of each recombinant MVA, respectively. Sera were collected at the indicated time point to detect NA against pseudovirus of the SARS-CoV-2 ancestral strain, Delta strain, or Omicron BA.2 strains. Data are presented as mean ± SD (*n* = 5) and were normally distributed (Shapiro–Wilk, *p* > 0.05). Significant differences between groups were determined by employing a two-way ANOVA followed by Tukey’s multiple comparison test. ** *p* < 0.01.

Two groups of minks or cats were orally inoculated with 5 × 10^8^ TCID_50_ of MVA or the trivalent vaccine containing 5 × 10^8^ TCID_50_ of each recombinant MVA, respectively. We then tested the NA titers against SARS-CoV-2 pseudoviruses from the sera of the minks and cats. As shown in Figure 6A,B, SARS-CoV-2 NAs were detected in the blood of minks and cats inoculated with the trivalent vaccine, but not MVA. At three weeks p.v., the mean titers of the NA against ancestral, Delta, and Omicron strains for vaccinated minks were 5.05 log_2_, 5.75 log_2_, and 4.95 log_2_, and then reached a peak of 7.95 log_2_, 7.15 log_2_, and 6.8 log_2_ at two weeks after the second dose, respectively. At nine weeks p.v., the mean titers of SARS-CoV-2 NA for the group slowly declined to 7.20 log_2_, 6.65 log_2_, and 6.45 log_2_, respectively. No significant difference was observed in the SARS-CoV-2 NA titers for the group between the 5-week and 9-week time points p.v. In the experiment with cats, SARS-CoV-2 NA was detected in 3/5 vaccinated cats, but not in 2/5 vaccinated cats at 3 weeks p.v. (Figure 6B). Two weeks after boosting, the mean titers of NA against ancestral, Delta, and Omicron strains for the group reached 5.15 log_2_, 5.16 log_2_, and 5.36 log_2_, and then rose to 6.00 log_2_, 6.35 log_2_, and 6.30 log_2_ at 14 weeks p.v., respectively. No significant difference was observed in the SARS-CoV-2 NA titers for the group between the 5-week and 14-week time points p.v. All minks and cats remained healthy and exhibited no abnormal dietary behaviour or other clinical abnormalities during the immunization observation period. These results demonstrate that the trivalent vaccine is safe and orally immunogenic in minks and cats.

## 4. Discussion

The S protein, the primary target of SARS-CoV-2 neutralizing antibodies, is the preferred antigen for vaccine development. However, the wild-type S protein of SARS-CoV-2 exhibits poor immunogenicity and exists in metastable prefusion conformation [38,39]. Notably, a previous study confirmed that the S-6P variant, which incorporates six proline substitutions, demonstrates superior prefusion conformation stability, significantly enhanced expression levels, and markedly improved thermal stability compared to both the parental SARS-CoV-2 S protein and the S-2P variant [33].

In the present study, to optimize the immunogenicity of S protein, we designed and chemically synthesized three prefusion-stabilized S genes, S6P, DS6P, and BA2S6P, targeting the full-length S genes of the ancestral, Delta, and Omicron BA.2 strains of SARS-CoV-2. A mouse study demonstrated that the vaccination with DNA vaccines, expressing S6P, DS6P, or BA2S6P, individually or in combination, induces a strong and long-lasting NA response against homotypic SARS-CoV-2 pseudovirus. It indicated that these modified proteins are highly immunogenic in mice. Subsequently, we generated three recombinant MVAs (rMVA-S6P, rMVA-DS6P, and rMVA-BA2S6P) expressing S6P, DS6P, and BA2S6P, respectively, and evaluated their potential as vaccines against COVID-19. Animal studies demonstrated that these recombinant MVAs are immunogenic in mice, minks, and cats. More importantly, intramuscular administration of rMVA-S6P induced a strong NA response against live SARS-CoV-2 and completely protected mice against the SARS-CoV-2 challenge.

Susceptible animals, such as cats and minks, contribute significantly to the spread and evolution of SARS-CoV-2. Thus, developing effective strategies to prevent and control COVID-19 in these species is of considerable importance. However, developing COVID-19 vaccines specifically for animals has received comparatively little attention. Although several MVA-based COVID-19 vaccines have been reported to induced strong humoral and cellular immune responses in humans or animals [13,34,35,36,37,40,41,42,43], typically administered via intramuscular, subcutaneous, or intranasal routes. However, oral immunization offers an alternative approach that can boost vaccination coverage in animals, particularly in free-roaming animals and wildlife. Decades of global rabies control efforts demonstrate that oral vaccine delivery is the most effective strategy for managing infectious diseases in stray, uncatchable, and wild animals. Our previous work showed that an oral rabies virus-vectored COVID-19 vaccine candidate elicited high levels of SARS-CoV-2 neutralizing antibodies and effectively prevents infection and transmission in mice and minks [44]. Nevertheless, MVA offers superior safety characteristics as a live viral vector compared to RABV. Therefore, we further evaluated the oral immunogenicity and protective efficacy of rMVA-S6P in mice. Our results demonstrate that rMVA-S6P is highly immunogenic via the oral route and confers complete protection against the SARS-CoV-2 challenge. This protective efficacy can be attributed to the ability of oral vaccination to elicit simultaneous systemic and mucosal humoral and cellular immune responses, thereby offering broader and more durable protection [45]. These findings indicate that rMVA-S6P is a promising oral COVID-19 vaccine candidate and may inform novel approaches to developing vaccines against other infectious diseases.

SARS-CoV-2 has been undergoing rapid evolution, and several strains, such as the ancestral strain and variants Delta and Omicron, are frequently co-circulating. Moreover, there are obvious differences in the antigenicity among some virus strains, resulting in limited cross-variant protection [46]. It makes the prevention and control of COVID-19 even more difficult. Therefore, we further investigated the potential of combining these recombinant viruses as a COVID-19 trivalent vaccine. Our results demonstrated that oral or intramuscular vaccination with the trivalent vaccine is safe and induces a robust, sustained neutralizing antibody response against the SARS-CoV-2 pseudovirus in mice, minks, and cats. The titers of SARS-CoV-2 NA for both orally and intramuscularly immunized mice decreased very slowly after reaching the peak, remaining above 6.75 log_2_ and 8.25 log_2_ at 54 weeks p.v., respectively. These findings indicated that combined immunization is an effective administration strategy for the MVA-vectored COVID-19 vaccines.

For recombinant vaccine viruses, a paramount concern is that the insertion of a foreign gene should not increase the virulence of the viral vector. The S protein of SARS-CoV-2 serves not only as a key protective antigen but also as a major virulence factor. Our results demonstrated that the expression of the modified S proteins did not elevate the pathogenicity of the MVA vector in mice. This is likely attributable to the inherent replication deficiency of MVA in human and most mammalian cells, resulting only in transient infection. In fact, all mice, minks, and cats remained healthy and exhibited no abnormal dietary behaviour or other clinical abnormalities during the immunization observation period.

Overall, the three recombinant MVAs represent promising COVID-19 vaccine candidates for minks and cats. However, their safety, efficacy, and duration of immunity across varying ages and immunological backgrounds in these species remain to be elucidated through comprehensive animal trials.

## 5. Conclusions

This study developed three recombinant MVAs (rMVA-S6P, rMVA-DS6P, and rMVA-BA2S6P) designed to express the prefusion-stabilized S proteins S6P, DS6P, and BA2S6P, which correspond to the S proteins of the ancestral, Delta, and Omicron BA.2 strains of SARS-CoV-2, respectively. These recombinant MVAs are safe and immunogenic in mice, minks, and cats. The administration of these recombinant viruses, individually or in combination via oral or intramuscular routes, elicits robust and durable humoral immune response and provides complete protection against SARS-CoV-2 in mice, minks, and cats. These findings indicate that MVA-vectored vaccines are promising multi-route administered COVID-19 vaccine candidates for minks and cats.

## Data Availability

Data are contained within the article.
